# Biofunctionalized CdS Quantum Dots: A Case Study on Nanomaterial Toxicity in the Photocatalytic Wastewater Treatment Process

**DOI:** 10.1021/acsomega.3c00496

**Published:** 2023-05-22

**Authors:** Kavitha Shivaji, Kishore Sridharan, D. David Kirubakaran, Jayaramakrishnan Velusamy, Seyedeh Sadrieh Emadian, Satheesh Krishnamurthy, Anitha Devadoss, Sanjay Nagarajan, Santanu Das, Sudhagar Pitchaimuthu

**Affiliations:** † Department of Biotechnology, K. S. Rangasamy College of Technology, Tiruchengode 637215, India; ‡ Department of Nanoscience and Technology, School of Physical Sciences, University of Calicut, Thenhipalam 673635, India; § Department of Physics, K. S. R College of Arts and Science for Women, Tiruchengode 637215, India; ∥ Department of Chemical Engineering and Biotechnology, University of Cambridge, Philippa Fawcett Drive, Cambridge CB3 0AS, U.K.; ⊥ School of Engineering and Innovation, The Open University, Milton Keynes MK7 6AA, U.K.; # Institute of Biological Chemistry, Biophysics and Bioengineering (IB3), School of Engineering and Physical Sciences, Heriot-Watt University, Edinburgh EH14 4AS, U.K.; ¶ Department of Chemical Engineering, University of Bath, Bath BA2 7AY, U.K.; ∇ Department of Ceramic Engineering, Indian Institute of Technology (BHU), Varanasi 221005, India; ○ Research Centre for Carbon Solutions, Institute of Mechanical, Processing and Energy Engineering, School of Engineering and Physical Sciences, Heriot-Watt University, Edinburgh EH14 4AS, U.K.

## Abstract

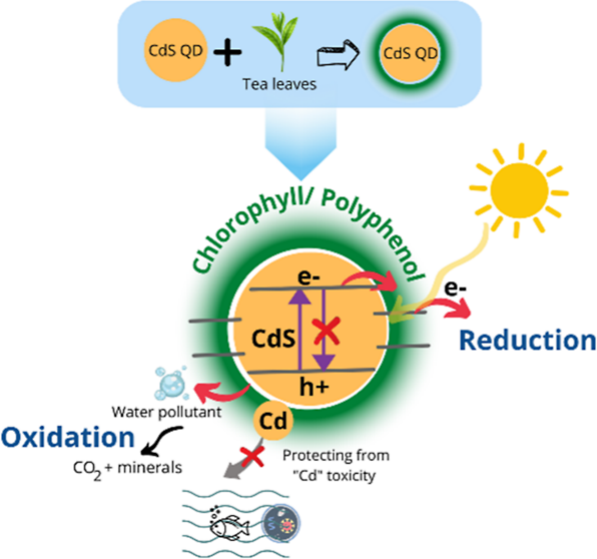

The toxic nature of inorganic nanostructured materials as photocatalysts is often not accounted for in traditional wastewater treatment reactions. Particularly, some inorganic nanomaterials employed as photocatalysts may release secondary pollutants in the form of ionic species that leach out due to photocorrosion. In this context, this work is a proof-of-concept study for exploring the environmental toxicity effect of extremely small-sized nanoparticles (<10 nm) like quantum dots (QDs) that are employed as photocatalysts, and in this study, cadmium sulfide (CdS) QDs are chosen. Typically, CdS is an excellent semiconductor with suitable bandgap and band-edge positions that is attractive for applications in solar cells, photocatalysis, and bioimaging. However, the leaching of toxic cadmium (Cd^2+^) metal ions due to the poor photocorrosion stability of CdS is a matter of serious concern. Therefore, in this report, a cost-effective strategy is devised for biofunctionalizing the active surface of CdS QDs by employing tea leaf extract, which is expected to hinder photocorrosion and prevent the leaching of toxic Cd^2+^ ions. The coating of tea leaf moieties (chlorophyll and polyphenol) over the CdS QDs (referred to hereafter as G-CdS QDs) was confirmed through structural, morphological, and chemical analysis. Moreover, the enhanced visible-light absorption and emission intensity of G-CdS QDs in comparison to that of C-CdS QDs synthesized through a conventional chemical synthesis approach confirmed the presence of chlorophyll/polyphenol coating. Interestingly, the polyphenol/chlorophyll molecules formed a heterojunction with CdS QDs and enabled the G-CdS QDs to exhibit enhanced photocatalytic activity in the degradation of methylene blue dye molecules over C-CdS QDs while effectively preventing photocorrosion as confirmed from cyclic photodegradation studies. Furthermore, detailed toxicity studies were conducted by exposing zebrafish embryos to the as-synthesized CdS QDs for 72 h. Surprisingly, the survival rate of the zebrafish embryos exposed to G-CdS QDs was equal to that of the control, indicating a significant reduction in the leaching of Cd^2+^ ions from G-CdS QDs in comparison to C-CdS QDs. The chemical environment of C-CdS and G-CdS before and after the photocatalysis reaction was examined by X-ray photoelectron spectroscopy. These experimental findings prove that biocompatibility and toxicity could be controlled by simply adding tea leaf extract during the synthesis of nanostructured materials, and revisiting green synthesis techniques can be beneficial. Furthermore, repurposing the discarded tea leaves may not only facilitate the control of toxicity of inorganic nanostructured materials but can also help in enhancing global environmental sustainability.

## 1 Introduction

The impact of the groundbreaking discovery of the photoelectrocatalytic water oxidation process at the semiconductor surface, popularly known as the Fujishima–Honda effect, has accelerated the photocatalysis (PC) technology for many applications that are beyond fuel (hydrogen and oxygen) generation.^1^ For instance, the elimination of air pollutants,^2,3^ wastewater treatment systems,^4−6^ and self-cleaning coatings^7,8^ are some of the highly impactful applications of PC technology in the industrial market. In PC reactions, light illumination is a key factor that induces the production of the photogenerated charge carriers (e^–^ and h^+^) in the semiconductor for driving the oxidation and reduction reactions. On the other hand, semiconductor bandgap energy (in eV) equivalent to the normal hydrogen electrode (NHE in V) dictates the PC reaction rate. Typically, a semiconductor photocatalyst with a valence band (VB) position equal to or greater than 0.8 V can produce hydroxyl radicals (OH^•^), while superoxide radicals (O_2_^–•^) are produced only when the conduction band (CB) is lower than −0.34 V. Organic contaminants adsorbed onto the surface of a photocatalyst are easily oxidized by the energetic OH^•^ radicals and converted into CO_2_ and minerals. Similarly, the O_2_^–•^ produced at the CB are converted to OH^•^ with the formation of hydrogen peroxide as an intermediate that can eliminate water and airborne pathogens (bacteria, virus, fungus, etc.) and cancer cells. Therefore, the effective generation, subsequent separation, and utilization of the electron–hole pairs dictate the efficiency of a photocatalyst.

Particle size is another crucial factor that determines the performance of particulate photocatalysts.^9,10^ In general, nanoscale photocatalysts exhibit enhanced activity compared to the bulk owing to their large surface area-to-volume ratio, which has been well documented in the literature. Typically, nanoparticles of TiO_2_, CeO_2_, and ZnO with mean sizes of 25–30 nm have been reported as ideal photocatalysts for industrial applications that are attributed to their biocompatible nature in addition to their excellent photocatalytic activity.^11,12^ Recently, the sustainable model targeting sunlight-driven activity and low operational cost is getting profound attention as solar energy is abundant and does not negatively affect the environment. Practically, a significant fraction (∼55%) of the sunlight is available in the visible wavelength region (400–800 nm), but unfortunately, successful traditional photocatalysts like TiO_2_ are operational only under the UV-light region owing to their large bandgap. Thus, nanoscale semiconductors with a narrow bandgap energy (1.5–2.5 eV) are ideal for efficiently harvesting photons from sunlight.^13,14,54^

In this regard, a variety of nanoscale semiconductors with narrow bandgap energy, including metal oxides (BiVO_4_, Fe_2_O_3_, CuO, and WO_3_),^15−17^ metal chalcogenides (CdS, CdSe, CuInS, and PbS),^18−21^ carbonaceous materials (graphene and its composite),^22,23^ and polymer-like materials (g-C_3_N_4_),^24,25^ have been developed as photocatalysts for treatment of pollutants in wastewater and for producing hydrogen. Among these, semiconductor quantum dots (QDs) with sizes between 1 and 10 nm exhibit great potential in harvesting sunlight over a broad spectral range due to their large extinction coefficients and high surface-to-volume ratios. Despite the tunable surface chemistry of QDs, incorporating a stabilizing agent or linker molecules (organic ligands),^26^ is often a challenge as these chemicals are expensive and potentially toxic to the environment. Moreover, the prolonged light illumination over the QDs during PC reactions can trigger photocorrosion and leads to the leaching of the constituent elements into the reaction medium that could be toxic. For instance, CdS QDs can favorably support almost all PC reactions owing to their favorable VB and CB positions and good visible-light absorption.^27,28^ However, under prolonged light exposure, the CdS QDs undergo photocorrosion due to the reaction with photogenerated holes, which destroys their structural stability and causes their disintegration into Cd^2+^ and S^–^. A similar issue of photocorrosion is reported on other semiconductor photocatalysts such as ZnO and CuO.^29−31^ Generally, an inorganic passivation layer of ZnS, NiP,^32^ or amorphous metal oxides (TiO_2_, ZrO_2_, Cr_2_O_3_, etc.) is used for wrapping the QDs in a core–shell fashion to protect them from photocorrosion by the effective entrapment of the photogenerated holes at the QDs/passivation layer interface. However, the light-blocking effect and charge transfer resistance caused by the shell layer can negatively affect photocatalytic performance.

Comparatively, bioderived materials (plant leaf, biomass, algae, fungus, etc.)^33−35^ offer several advantages as a shell layer^36^ by facilitating good light-harvesting properties and enhanced catalytic activity through charge separation. Furthermore, the bioderived materials also act complementary to the surface of the nanoscale photocatalysts as they can alleviate the toxicity effect expected due to photocorrosion. Green synthesis of nanoscale materials using plant extract has attracted a great deal of attention in recent years owing to the advantage of avoiding the usage of environmentally unfriendly organic solvents. A range of environmentally friendly phytochemicals present in the plant extract include polyphenols, vitamins, sugars, proteins, polysaccharides, sterols, triterpenes, alkaloids, and amino acids, antioxidant metabolites and flavonoids act as particle-stabilizing or metal-reducing agents.^37,38^ Hence, phytochemical-mediated synthesis of nanoscale semiconductors is expected to give environmentally friendly photocatalysts. Chlorophyll, the green pigment in plants that initiates the photosynthesis process by absorbing sunlight, is often studied owing to its high fluorescence quenching capability when it is conjugated with metal nanoparticles.^39−42^ Core–shell structure formation between chlorophyll and semiconductor nanoparticles is expected to inhibit the photogenerated charge carriers formed in the core and the shell for enhancing the charge separation. Though significant studies on the synthesis of chlorophyll/metal nanoparticles are available in the literature, the fabrication of chlorophyll-functionalized semiconductor nanomaterials is rarely reported.

**Figure 1:**
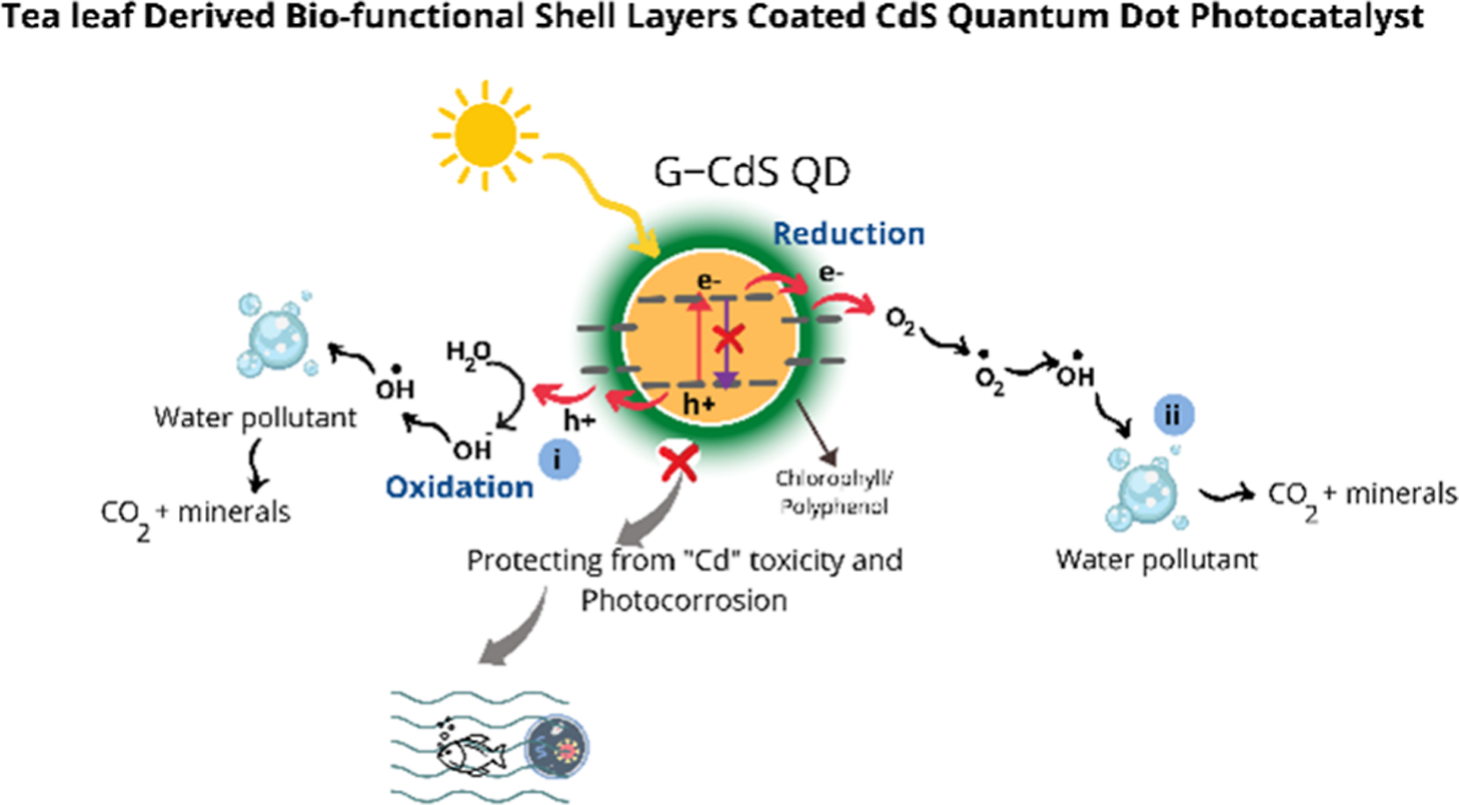
Schematic illustration of photocorrosion of CdS QDs and the subsequent leaching of toxic Cd^2+^ ions into freshwater sources and advantages of tea leaf-mediated CdS QDs in enhancing the transfer of the photogenerated charge carriers and protecting the core CdS QDs from photocorrosion.

As depicted in Figure 1, this work demonstrates the advantages of tea leaf extract-mediated synthesis of CdS QDs (referred to hereafter as G-CdS QDs). Interestingly, a layer of chlorophyll/polyphenol coated over the G-CdS QDs promoted the quick transfer of photogenerated charge carriers and enabled enhanced photocatalytic activity while protecting the core CdS QDs from photocorrosion, thereby ensuring that the toxic Cd^2+^ ions do not enter the environment. Structural, morphological, optical, and biocompatibility properties of G-CdS QDs are compared with those synthesized through a conventional chemical synthesis approach (referred to hereafter as C-CdS QDs). Compared to the typical reports on the photocatalytic activity of nanostructured photocatalysts, this study also focuses on accessing their environmental impact, which is seldom explored. Toward this end, zebrafish embryos were treated with the as-synthesized CdS QDs for realizing their toxicity and biocompatibility.

## 2 Experimental Section

Cadmium sulfate (CdSO_4_, 99.99% purity) and sodium sulfide (Na_2_S, 98% purity) were purchased from Merck India and were used as received without purifying further. Deionized water from the ultrapure water purification system was used throughout the experiments.

### 2.1 CdS QD Synthesis

Fresh tea leaves obtained from a tea plant were thoroughly washed with deionized water and dried under shade. Next, the leaves were carefully chopped and incubated for 24 h after mixing them with methanol (1 g/10 mL ratio). After incubation, the leaf extract solution was filtered through Whatman qualitative filter paper (grade number 1) and safely stored in a refrigerator (4 °C) for further use. CdS QDs using the prepared tea leaf extract (*Camellia sinensis*) were synthesized by following the procedure reported by us previously.^43,44^ Typically, a solution was prepared by adding 0.025 M CdSO_4_ to 10 mL of the prepared *C. sinensis* extract and was incubated in the dark for a few days. Next, a freshly prepared 0.025 M Na_2_S solution was added to the prepared CdSO_4_ solution, and this final solution was incubated further for few more days for yielding CdS QDs. A longer incubation period resulted in a larger particle size, and in this work, we kept it for 7 days. The resulting solution was centrifuged repeatedly with deionized water, and the bright-yellow sediments retrieved after drying were named G-CdS QDs, where the letter “G” denotes green. For comparative studies, conventional CdS QDs were synthesized through the same procedure without the addition of tea leaf extract and the resultant particles were named C-CdS QDs, where C represents chemical.

### 2.2 Characterization

The structural properties of the as-synthesized CdS QD powder samples were analyzed using X-ray diffraction (XRD, Bruker D8 Discover, Germany) with Cu Kα radiation (λ = 1.5418 Å) at a scan rate of 2°/min in the 2θ range of 10–80°. Low- and high-resolution transmission electron microscopy (HRTEM) analyses were performed using a TECNAI (G2 20 TWIN, F.E.I., United States of America) microscope operated at an acceleration voltage of 200 kV. Samples for TEM analysis were prepared by dropwise addition of a few drops of the CdS QD solution dispersed in ethanol onto a lacy carbon-coated copper grid. X-ray photoelectron spectroscopy (XPS) was carried out in a PREVAC EA15 system equipped with a 180° electrostatic hemispherical analyzer, a 7 multichannel detector, and two multichannel plates, using a monochromatic Al Kα radiation (1486.6 eV) operated at 12 kV and 25 mA X-ray source. The survey spectra were taken between 0 and 1200 eV with both survey and high-resolution scans recorded at a pass energy of 100 eV. Electron charge neutralization was achieved using a PREVAC flood source FS40-PS with an ion gun current of 3 μA and an ion gun voltage of 0.2 V. All sample data was recorded at a pressure below 10–9 mPa. UV–vis absorption spectra of the as-synthesized CdS QDs dispersed in ethanol were recorded on a spectrophotometer (PerkinElmer, Lambda 365, United Kingdom). Photoluminescence spectra were recorded on a spectrofluorometer (Horiba, FluoroMax-4) by dispersing the synthesized CdS QDs in dimethyl sulfoxide. To ensure good optical quality, the concentration was carefully optimized to an optical density value of 0.2. The PL lifetime decay measurements were performed under the time-correlated single-photon counting (TCPSC) mode on a spectrofluorometer (Edinburg Instruments, FS5) with a 1 cm × 1 cm quartz cell. In TCPSC measurement, a picosecond (ps) pulsed laser of 405 nm (3.06 eV) with a repetition rate of 1 MHz was used as the excitation source. Both prompt (IRF) and sample measurements were performed with less than 5% fluorescence signals to avoid pulse pile-up issues in the photomultiplier tube.

### 2.3 PC Experiments

Photocatalytic activity of the as-synthesized CdS QDs was assessed by monitoring the degradation of the methylene blue (MB) dye solution under visible-light (λ > 400 nm) irradiation at room temperature. Visible light-driven photodegradation reactions were carried out using a photocatalytic reactor constructed in-house that was powered by a 150 W tungsten halogen lamp.^45^ Typically, 25 mg of the CdS QD photocatalyst was suspended in 50 mL of an MB dye solution (10 ppm) for preparing the reaction slurry. Next, the slurry was stirred in the dark condition for 30 min to ensure MB dye adsorption on the surface of CdS QDs. The PC reaction was initiated by switching on the lamp, and the distance between the source and the slurry was noted to be 6 cm. The solution was exposed to visible-light irradiation with continuous air purging and magnetic stirring to facilitate agitation. The UV–vis absorption spectrum of the aliquots (4 mL) withdrawn at every 10 min interval after centrifugation and filtration was recorded using a UV–vis spectrophotometer. For consistency, the experiments were repeated two more times under similar conditions. For investigating the stability of the CdS QDs against photocorrosion, the experiments were repeated by reusing the photocatalyst after washing and drying at 60 °C.
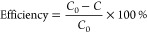


The degradation efficiency of MB was estimated using the given equation,^46^ where *C*_0_ is the initial MB dye concentration and *C* is the concentration of the MB dye after light irradiation. The Langmuir–Hinshelwood (L–H) model was preferred as the photocatalyst obeys pseudo-first-order kinetics. The kinetic plot was drawn using the relation
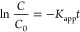
where *K*_app_ represents the apparent rate constant determined from the slope of the kinetic plot and *t* is the irradiation time.^47^

### 2.4 Zebrafish Maintenance and Embryo Harvesting

Matured zebrafish were procured from local sources and nurtured following the standard fish breeding protocol. Adult zebrafish were fed with commercially available protein pellets twice a day, and the water temperature was maintained at 28 °C with an alternative light/dark photocycle. Adult males and females of zebrafish in a 2:1 ratio were chosen and placed in a breeding tank overnight for spawning. On the following day early morning, spawning was induced with the onset of white light. The embryos were collected and carefully transferred within 30 min to a Petri dish containing the E3 (embryo medium: 5 mM NaCl, 0.17 mM KCl, 0.33 mM CaCl_2_, 0.33 mM MgSO_4_) medium for further analysis.

Zebrafish embryos were separated randomly (*n* = 15) with the E3 medium before treating them with as-synthesized CdS QDs until the hatching stage as per the OECD guidelines 210. Toxicity studies were conducted by monitoring the growth of the embryos under a stereomicroscope (Leica M 165 FC Fluorescence Stereo Microscope with Digital Camera; Leica Microsystems Vertrieb Gmbh, Germany, M 165 FC) at specific time intervals (24, 48, and 72 hpf) after exposing them to the synthesized CdS QDs of various concentrations (10, 20, 30, 40, and 50 μg/mL). Developmental changes (survival rate, heart rate, hatching rate, tail deformities, yolk sac edema, and eye deformities) observed in the treated embryos were compared with untreated embryos (control).

## 3 Results and Discussion

**Figure 2:**
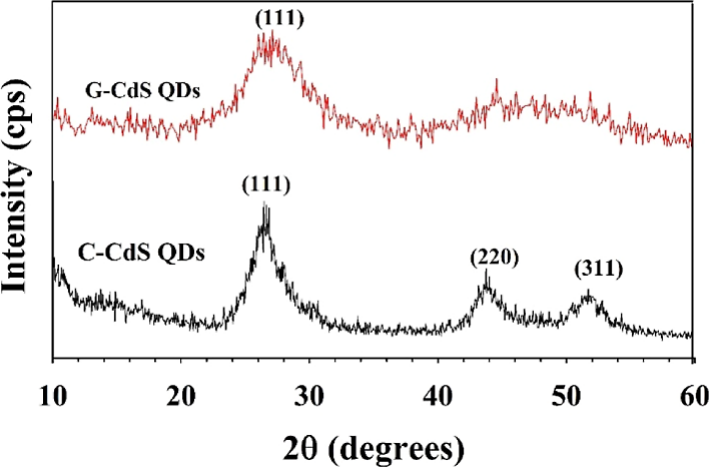
Comparative XRD patterns of the as-synthesized G-CdS QDs (red) and C-CdS QDs (black). Both patterns match well with the fcc structure of CdS. However, the influence of polyphenol/chlorophyll from the tea leaf extract is evident from the subdued XRD pattern of G-CdS QDs.

The crystal structure of the as-synthesized CdS QDs was determined through powder XRD, and the corresponding patterns are shown in Figure 2. XRD patterns of C-CdS QDs without tea leaf extract exhibited broad peaks at 2θ = 26.4, 43.7, and 51.8°, which match well with the (111), (220), and (311) planes of the face-centered cubic (fcc) structure of CdS (JCPDS 89-0440).^48,49^ Comparatively, the diffraction patterns of G-CdS QDs exhibited a single broad peak centered at 2θ = 26.5° that could be indexed to the (111) plane of cubic CdS. Other peaks in G-CdS QDs were found to be overlapped and not clear, which could be attributed to the influence of polyphenol/chlorophyll coated over the surface of CdS QDs, similar to those reported previously.^43,44^ Typically, the incubation of CdSO_4_ in tea leaf extract for 3 days resulted in the binding of negatively charged polyphenol/chlorophyll over the surface of Cd^2+^ ions.^50^ Furthermore, the S^2–^ ions (after adding the sulfur precursor) slowly interacted with Cd^2+^ ions to form CdS QDs, which comparatively was much faster in the case of C-CdS QDs. Therefore, the polyphenol molecules present on the surface of Cd^2+^ influenced the particle size and retarded the rate of nuclei formation in G-CdS QDs. Also, the Brownian motion within the reaction medium was poor, and correspondingly, the Ostwald ripening was inefficient to produce more nuclei as the synthesis of G-CdS QDs was conducted at room temperature.^51^

**Figure 3:**
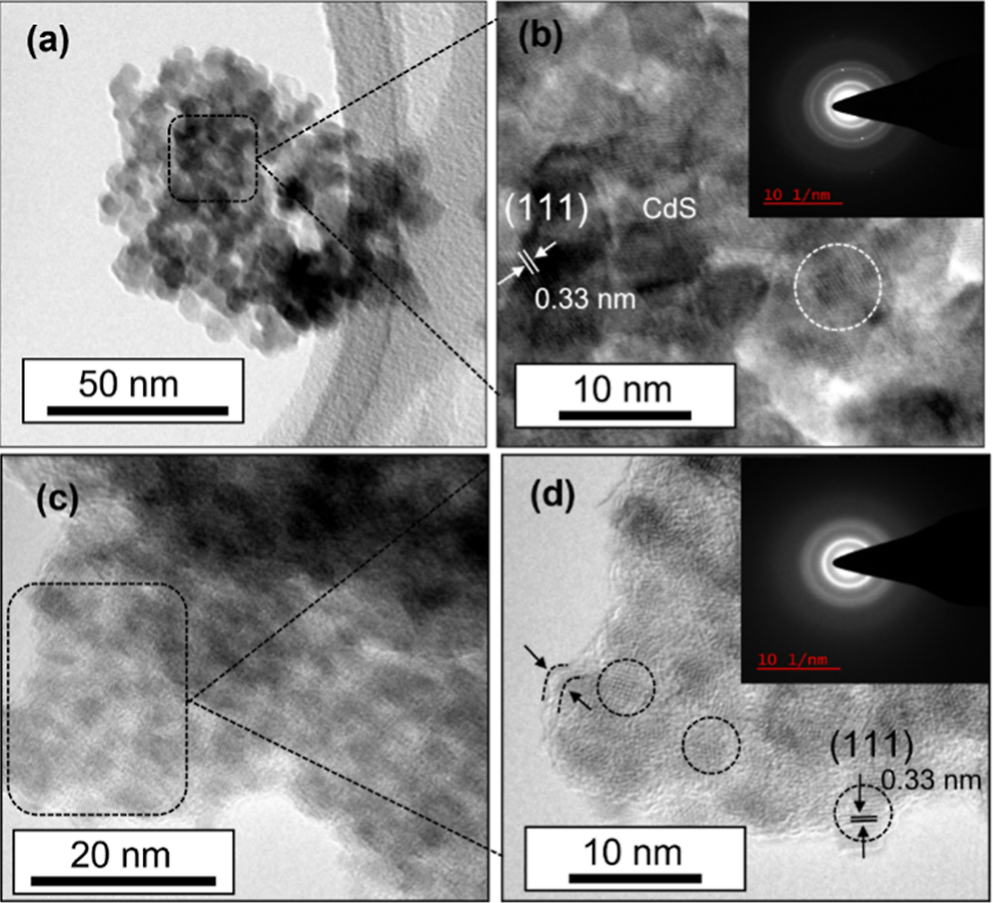
TEM micrographs of the as-synthesized (a) C-CdS QDs and (c) G-CdS QDs. HRTEM micrographs of (b) C-CdS QDs and (d) G-CdS QDs revealing the fringe spacing of 0.33 nm corresponding to the (111) plane of cubic CdS. The presence of a polyphenol/chlorophyll layer over the surface of G-CdS QDs can be observed from the HRTEM micrograph marked in (d). SAED patterns shown in the insets of (b,d) indicate the polycrystalline nature of the as-synthesized CdS QDs.

TEM analysis of the as-synthesized CdS QDs presented in Figure 3 was performed to counter-verify the size and crystal structure. As observed from Figure 3a, the average size of C-CdS QDs was 6–8 nm and the corresponding HRTEM micrograph in Figure 3b indicated the uniformly spread crystalline lattice fringes with a *d*-spacing of 3.3 Å, which matches well with the (111) plane of fcc CdS. On the other hand, as observed from Figure 3c, the average size of G-CdS QDs is much smaller (∼2–4 nm). Similarly, the HRTEM micrograph depicted in Figure 3d revealed the presence of lattice fringes with a *d*-spacing of 3.3 Å, which also clearly corresponded to the (111) plane of fcc CdS. However, the HRTEM micrograph in Figure 3d distinctly indicates the presence of a polyphenol/chlorophyll layer on the G-CdS QDs (indicated by the portion marked in between the arrows on the left side of Figure 3d). Furthermore, in comparison to the sharp and bright rings with some spots on the selected-area electron diffraction (SAED) pattern of C-CdS QDs (inset of Figure 3b), the intensities of the rings in the SAED pattern of G-CdS QDs (inset of Figure 3d) are dull and do not contain any spots that are indicative of their poor crystallinity, which is consistent with the XRD results. Moreover, the presence of polyphenol/chlorophyll was confirmed in our earlier work^43^ through Fourier transform infrared spectroscopy. The chemical linkage between the CdS QDs and the phytochemicals from the tea leaves was also confirmed in our earlier work on tea leaf-derived QD samples.^43^ Especially, the weak peak observed at ∼617 cm^–1^ could be attributed to the vibration related to metal sulfide, which confirmed the formation of CdS QDs. Similarly, the difference in the sharpness of the lattice fringes in the HRTEM micrographs of G-CdS QDs (Figure 3d) and C-CdS QDs (Figure 3b) is also indicative of the poor crystallinity of G-CdS QDs, which can again be attributed to the influence of the polyphenol/chlorophyll layer. However, both the lattice-resolved HRTEM micrographs explicitly illustrate the nearly parallel atomic planes with a fringe spacing of 0.33 nm corresponding to the (111) plane of fcc CdS, which are consistent with the XRD results.

**Figure 4:**
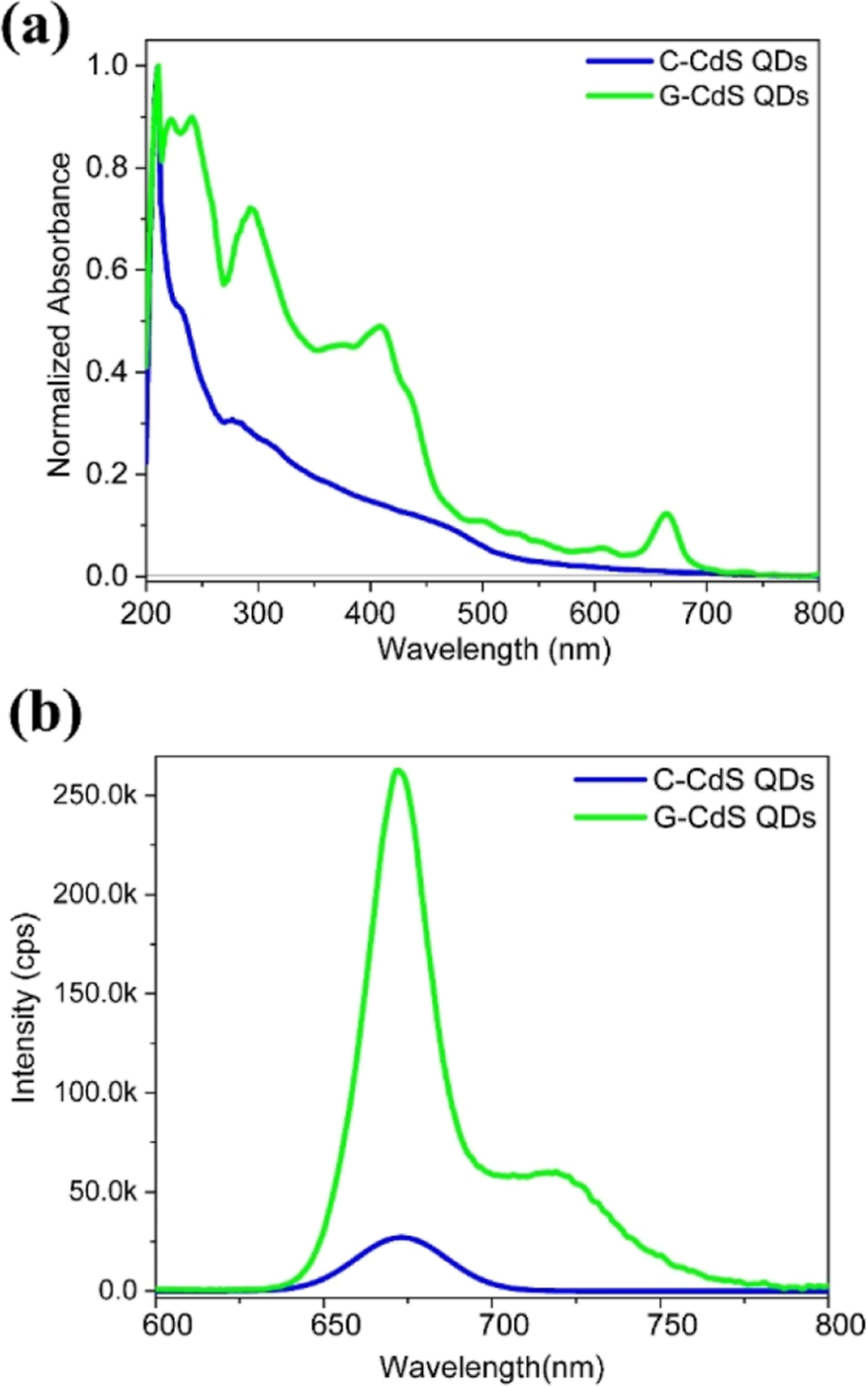
(a) UV–vis optical absorption and (b) room-temperature photoluminescence spectra of the as-synthesized G-CdS and C-CdS QDs. Significant variation in the absorption and photoluminescence spectra of G-CdS QDs in comparison to C-CdS QDs can be attributed to the polyphenol/chlorophyll layer coated over G-CdS QDs.

Studying the optical properties is critical for determining the capability of a material to function as a photocatalyst. UV–vis absorption spectra of the as-synthesized G-CdS and C-CdS QDs are shown in Figure 4a. Interestingly, G-CdS QDs have a significantly strong absorption across the UV and visible-light region in comparison to C-CdS QDs. The absorption band appearing at around 250 nm in G-CdS QDs can be attributed to π–π* transition of the C=C bonds due to the presence of polyphenols, and the weak absorption shoulder at 678 nm could be attributed to chlorophyll.^44,52^ Another significant absorbance peak observed around 410 nm was attributed to CdS QDs.^53^ Hence, polyphenol/chlorophyll moieties from tea leaf extracts not only function as size-controlling agents but are also responsible for the improved light-harvesting ability of the CdS QDs via the elevated reflection and refraction of light in the interior.^55^ The red shift in the absorption band edge of a photocatalyst is related to the transfer of the electron excitation energy between the conduction and VB,^56,57^ and therefore, the observed red shift in G-CdS QDs is expected to promote enhanced photocatalytic activity.^58^Figure 4b shows the room-temperature emission spectra collected by exciting the as-synthesized G-CdS QDs at 405 nm. A dominant peak centered at 673 nm can be observed for both C-CdS and G-CdS QDs. However, the emission intensity of G-CdS QDs was remarkably greater than that of C-CdS NPs. Furthermore, G-CdS QDs exhibited an additional emission peak centered at 720 nm. The enhanced emission intensity of G-CdS QDs could be attributed to the change in the reflection due to enhanced surface roughness aided by the presence of the polyphenol/chlorophyll layer and the reduced size of the G-CdS QDs that facilitate multiple scattering of photons.^59,60^ On the other hand, the strong chelating and electrostatic interaction between organic polyphenol/chlorophyll moieties and CdS QDs enables efficient electronic transition that in turn facilitates enhanced emission. Polyphenol/chlorophyll imposing a synergistic effect with CdS QDs creates a proportional electronic structure that reduces the distance between the electronic levels (the HOMO of polyphenols with the VB of CdS and the LUMO of polyphenols and the CB of CdS), enables the excitation of the electrons, and increases the emission intensity. Furthermore, the formation of this synergistic electronic band structure in G-CdS QDs enhances the separation of the photogenerated electron–hole pairs.

**Figure 5:**
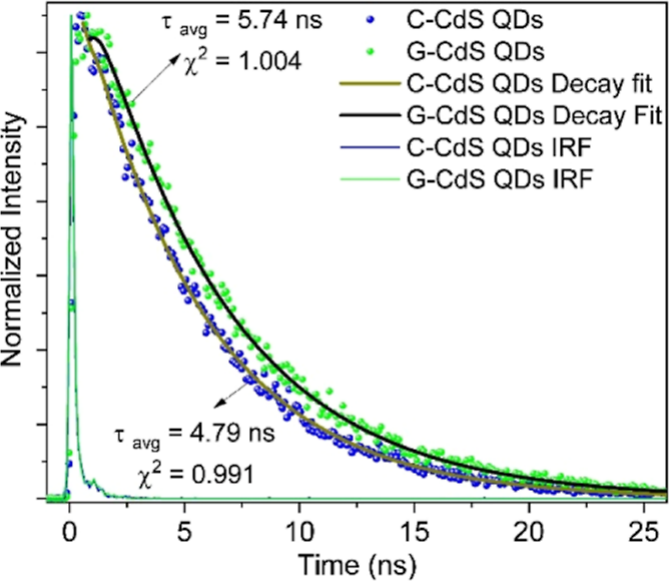
Time-resolved PL decay curves of G-CdS QDs and C-CdS NPs.

For gaining further insight into the charge-transfer kinetics, the PL decay spectra of the CdS NPs and G-CdS QDs were recorded at an excitation wavelength of 405 nm. Initially, the scattering contribution of the CdS QD samples was measured by the instrument response function (IRF). The recorded IRF data was used during the final data fitting for avoiding overestimation and error in the average lifetime values. The average lifetime (τ_ave_) value was calculated using the following equation
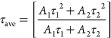
where τ_ave_ is the amplitude-weighted average lifetime of fluorescence, τ_1_ is the time constant of the fast decay process, τ_2_ is the time constant of the slow decay process, and *A*_1_ and *A*_2_ are the corresponding fractional values of the photoluminescence intensities. Figure 5 depicts the lifetime decay plots and τ_ave_ values of both samples along with their corresponding IRF curve. Polyphenol/chlorophyll containing G-CdS QDs exhibited an enhanced τ_ave_ of 5.74 ns, which indicated that the lifetime of the charge carriers was 20% greater than those of C-CdS NPs (τ_ave_ = 4.79 ns). An increase in the charge carrier lifetime in G-CdS QDs can be attributed to the delayed electron transfer through the polyphenol/chlorophyll layer and reduced nonradiative recombination. The χ^2^ value near unity indicates that both curve fitting and data taken for calculation are desirable.

### 3.1 Photocatalytic Activity Studies

**Figure 6:**
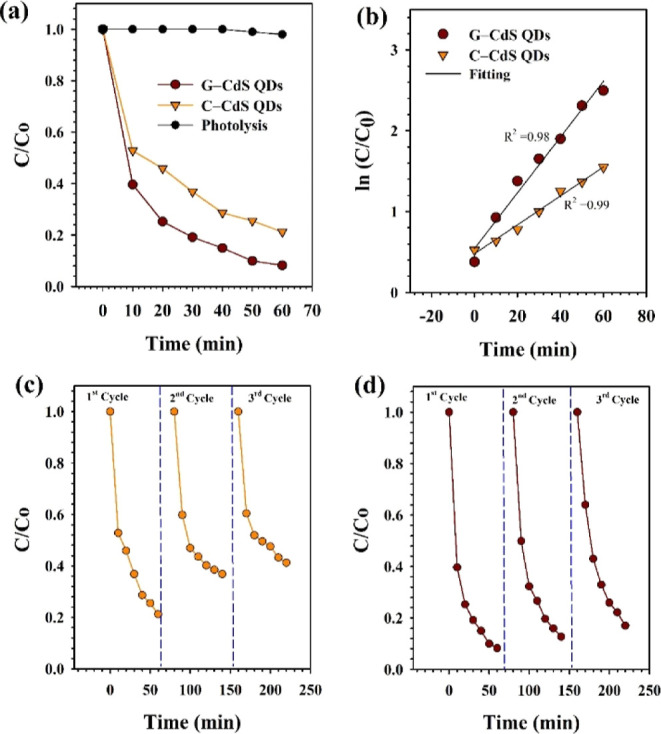
(a) Plot showing the time-dependent photocatalytic degradation of MB dye molecules and the corresponding (b) reaction rate constants under visible-light irradiation in the presence of G-CdS and C-CdS QDs. Recyclability test of (c) C-CdS QDs and (d) G-CdS QDs for three successive cycles of MB photodegradation under visible-light irradiation.

Visible-light-driven photocatalytic activity of the prepared G-CdS QDs was evaluated through the photocatalytic degradation of MB dye molecules in water. A plot showing the photocatalytic degradation of MB with respect to irradiation time is presented in Figure 6a. As observed from Figure 6a, the visible-light-driven photodegradation of MB was negligible in the absence of a photocatalyst, indicating that no photolysis occurred. Interestingly, the G-CdS QDs exhibited enhanced photocatalytic activity and degraded about 96% of MB dye within 60 min of visible-light irradiation, while C-CdS QDs could degrade only about 78% during the same irradiation time period.

As discussed earlier, the enhanced photocatalytic activity of G-CdS QDs can be attributed to their increased visible-light absorption and the synergistic electronic band structure resulting from the heterojunction between polyphenols and CdS QDs that facilitates improved separation of photogenerated charge carriers. Enhanced photodegradation was evident from the plot in Figure 6b, wherein the rate constant obtained using G-CdS QDs was significantly higher than that of C-CdS QDs. On the other hand, recyclability studies were conducted for understanding the role of polyphenol/chlorophyll in hindering photocorrosion of CdS QDs. Surprisingly, the presence of the polyphenol/chlorophyll layer in G-CdS QDs protected the core CdS QDs and imparted enhanced stability against photocorrosion. The plot showing the three consecutive cycles of photodegradation of MB dye molecules by recycling G-CdS QDs (Figure 6d) indicates the minimal decrease in the photodegradation efficiency in comparison to C-CdS QDs (Figure 6c). A marginal decrease in the photocatalytic activity of G-CdS QDs could be attributed to the loss of the photocatalyst during recycling. On the other hand, the poor photocorrosion stability of C-CdS QDs is due to the reaction of the photogenerated holes with CdS QDs, causing the leaching of toxic Cd^2+^ ions by disrupting the crystal structure as reported earlier.^60^

The stability of a photocatalyst employed for organic pollutant degradation from wastewater is a crucial factor since they are typically prone to severe chemical corrosion or photocorrosion. Chemical/photocorrosion of the photocatalyst leads to the leaching of metal ions as secondary pollutants into water, which can induce a potential environmental impact known as nanotoxicity that has severe implications for the aquatic environment and human health.^61^ Since the nanoparticle size (1–100 nm) is lesser than that of the biological cells, the possibility of subcellular interaction is very high.^62^ Therefore, recently, some review articles have focused on the toxicity and phototoxicity of nanoscale photocatalysts. One of the ways to overcome the nanotoxicity of photocatalysts is to passivate their surface by coating/coupling them with a chemically resistant material through chemical/physical methods.^63,64^ Another important way is to assess the nanotoxicity of the as-synthesized photocatalysts by making them react with some biological organisms such as zebrafish embryos.

### 3.2 Interaction of Biosynthesized Nanoparticles with Zebrafish Embryos

**Figure 7:**
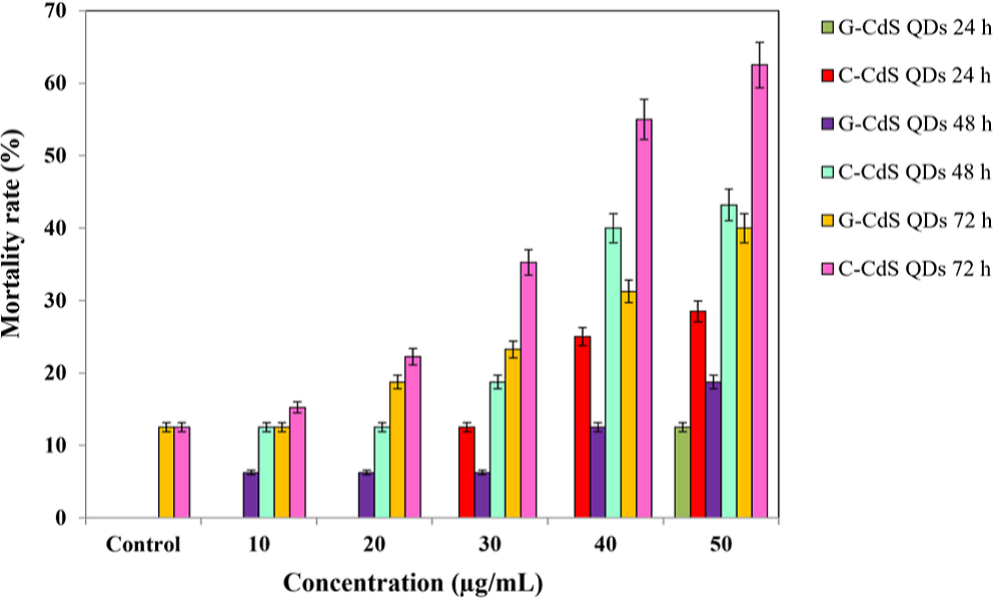
Estimated mortality rate of embryonic fishes at different concentrations of C-CdS QDs and G-CdS QDs.

The toxicological effect of the as-synthesized G-CdS QDs and C-CdS NPs was assessed by studying the survival rate, hatching rate, morphological variations, and phenotypic alteration in zebrafish. In-depth studies were conducted by varying the concentration of the G-CdS QDs (10–50 μg/mL) on the embryos for a maximum period of 72 h, and the results were compared with untreated embryos as a placebo. As observed from Figure 7, the survival rate at specific time points (24, 48, and 72 hpf) was assessed, and interestingly, both the G-CdS QDs and C-CdS NPs exhibited no toxic effect when the concentration was 10 μg/mL. Surprisingly, the mortality rate of embryos treated with 10–30 μg/mL G-CdS QDs was just 5% after 48 hpf, which doubled (10%) when the embryos were treated with C-CdS NPs. The mortality after 72 hpf with G-CdS QDs at 40 and 50 μg/mL concentrations was 31.25 and 40%, respectively. Comparatively, zebrafish embryos exposed to C-CdS NPs at the same concentrations of 40 and 50 μg/mL after 72 hpf were 55 and 62.25%, respectively, which was more than 1.5 times higher than that of G-CdS QDs. This result indicated that G-CdS QDs exhibited developmental toxicity in a time and dose-dependent manner that has a significantly less mortality rate in comparison to C-CdS NPs.

**Figure 8:**
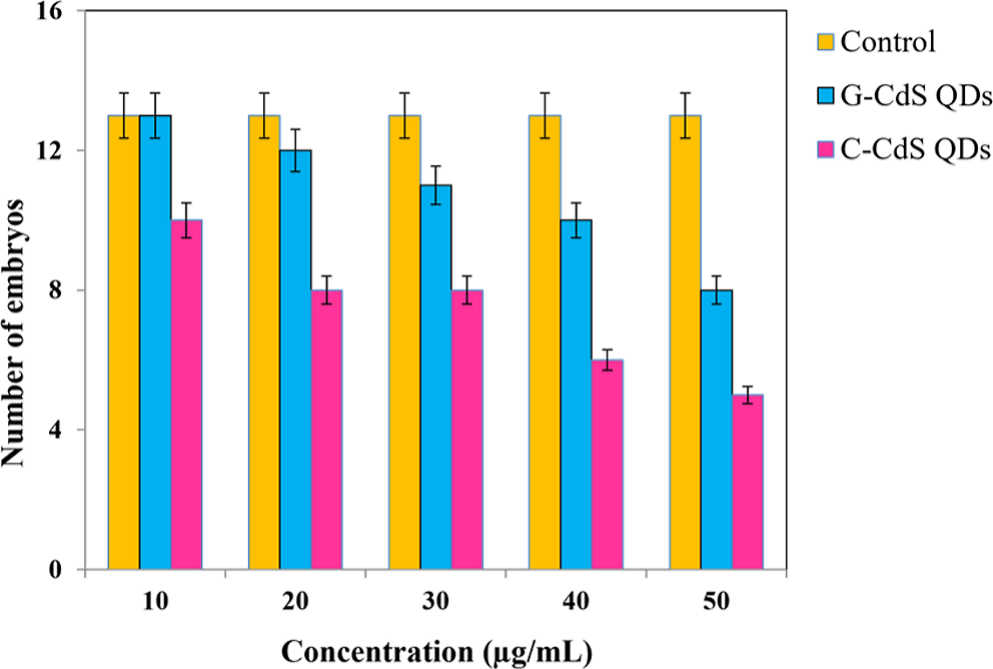
Estimated number of hatched embryonic fishes at different G-CdS QD concentrations and C-CdS QDs and compared with control.

Figure 8 depicts the influence of G-CdS QDs on the successful hatching of the zebrafish embryos. As observed, at a minimal concentration of 10 μg/mL, ∼13–14 embryos were successfully hatched in the presence of G-CdS QDs, which were equivalent to the control. Conversely, only ∼10 embryos were hatched at 10 μg/mL in the presence of C-CdS NPs. However, an increase in the concentration of the CdS QDs negatively affected the number of successful hatching of the embryos. Despite the negative influence, it is evident from the results that the toxicity effect of G-CdS QDs on the embryos was comparatively lesser than that of C-CdS NPs.

### 3.3 Malformations

**Figure 9:**
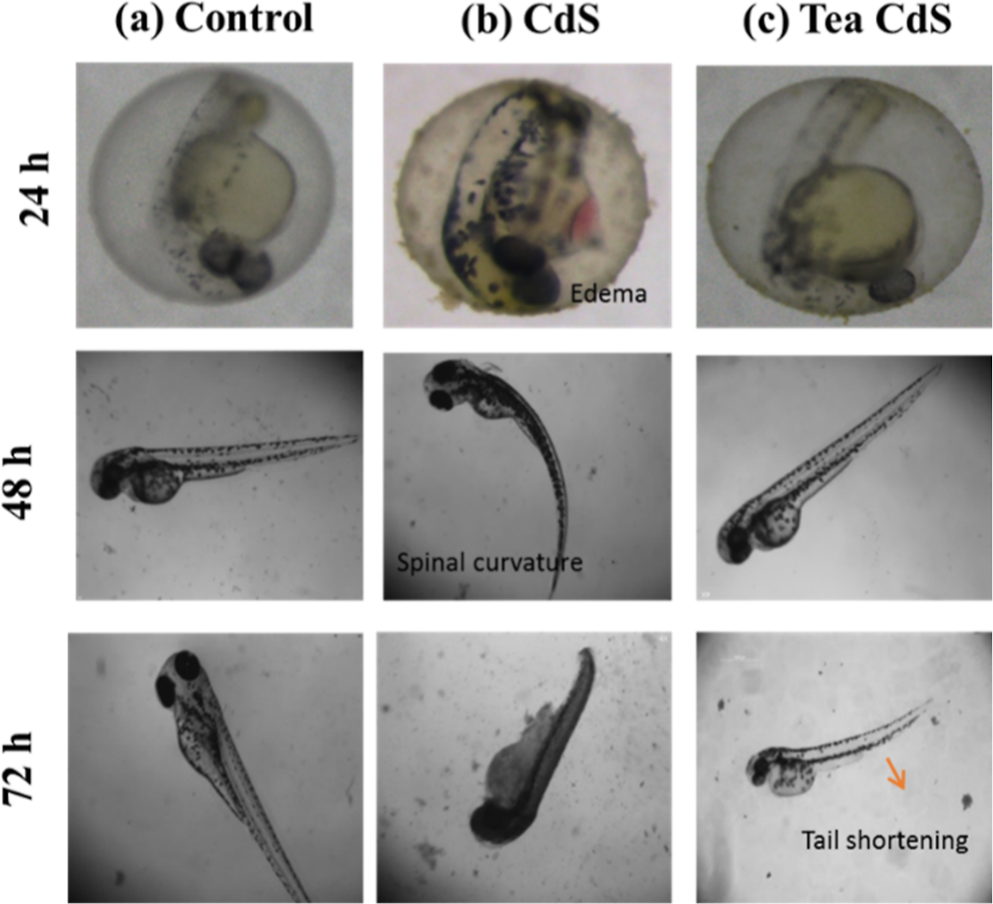
Microscopic images of (a) control embryos, (b) embryos treated with C-CdS QDs at 50 μg/mL, and (c) embryos treated with G-CdS QDs at a concentration of 50 μg/mL, observed at 24, 48, and 72 hpf, respectively.

Next, the malformations of the embryos treated with the as-synthesized G-CdS QDs at different stages (24, 48, and 72 hpf) were analyzed. As observed from Figure 9, the embryos treated with G-CdS QDs up to the larvae stage (24 hpf) did not exhibit any malformations at a concentration of 10 μg/mL (Figure S1b).

However, at higher concentrations of G-CdS QDs (30 and 50 μg/mL), the treated embryos exhibited bent tails, shortened tails, spinal curvature, and pericardial edema at 72 hpf **(**Figures S1b and 9c**)**. On the other hand, the embryos treated with high concentrations (30 and 50) of C-CdS QDs exhibited various defects (yolk sac edema, bent tail, shortened tail, spinal curvature, and pericardial edema) and eventually died **(**Figures S1a and 9b**)**. However, malformation (pericardial edema) was also observed in untreated embryos at 72 hpf **(**Figure 9a**)**. Therefore, conspicuously, the embryos treated with G-CdS QDs exhibited the least malformations even at higher concentrations than those treated with C-CdS QDs, which obviously can be attributed to the biocompatible shell layer containing the tea leaf moieties.

### 3.4 Delayed Hatching: Mechanism of Toxicity Exposure

This study observed the impact of G-CdS QDs on the development of zebrafish embryos. Typically, zebrafish embryos are demersal, and they tend to settle down at the bottom of the water column, which leads to their direct exposure to the sedimented particles. Therefore, the increased adherence of G-CdS QDs on the surface of the embryos at higher concentrations causes an abnormal physical effect, leading to toxicity.^63^ Apart from the malformation of the embryos, a delay in hatching was also observed. The probable mechanism causing the delay in hatching could be attributed to the aggregation of G-CdS QDs over the surface of the embryos, which modifies the surface mechanical properties that inhibit the function of the chorionic hatching enzyme.^65^ On the other hand, the accumulation of G-CdS QDs on the surface of the embryos can cause depletion of oxygen, leading to hypoxia, which results in delayed hatching.^65^ Another study suggested that excessive production of ROS under in vivo conditions could lead to the initiation of oxidative stress-induced developmental toxicity in embryos.^66^ Delayed hatching and several types of malformations in zebrafish embryos were reported when they were treated with CdTe QDs. It was revealed that embryonic toxicity including pericardial edema, yolk sac edema, and spinal curvature deformities caused by Cd^2+^ ions was dosage- and time-dependent.^67^ Similarly, our observations on the exposure of C-CdS QDs too are comparable to the study reported by Duan et al. on CdTe QDs.^67^ However, the G-CdS QDs with a biocompatible shell layer formed by tea leaf moieties play a significant role in protecting the zebrafish embryos from the toxic effect of Cd^2+^ ions. Furthermore, nullifying the nanotoxicity of nanostructured photocatalysts can enable them to be employed in biological applications. For example, CdS QDs coated with chlorophyll/polyphenol moieties have been found to be very efficient for in vivo imaging and drug carrier applications.^43,44^ In this context, we believe that this proof-of-concept study for assessing the nanotoxicity of nanostructured photocatalysts should be useful to the community of researchers working on PC.

### 3.5 Catalyst Stability before and after PC Reactions

**Figure 10:**
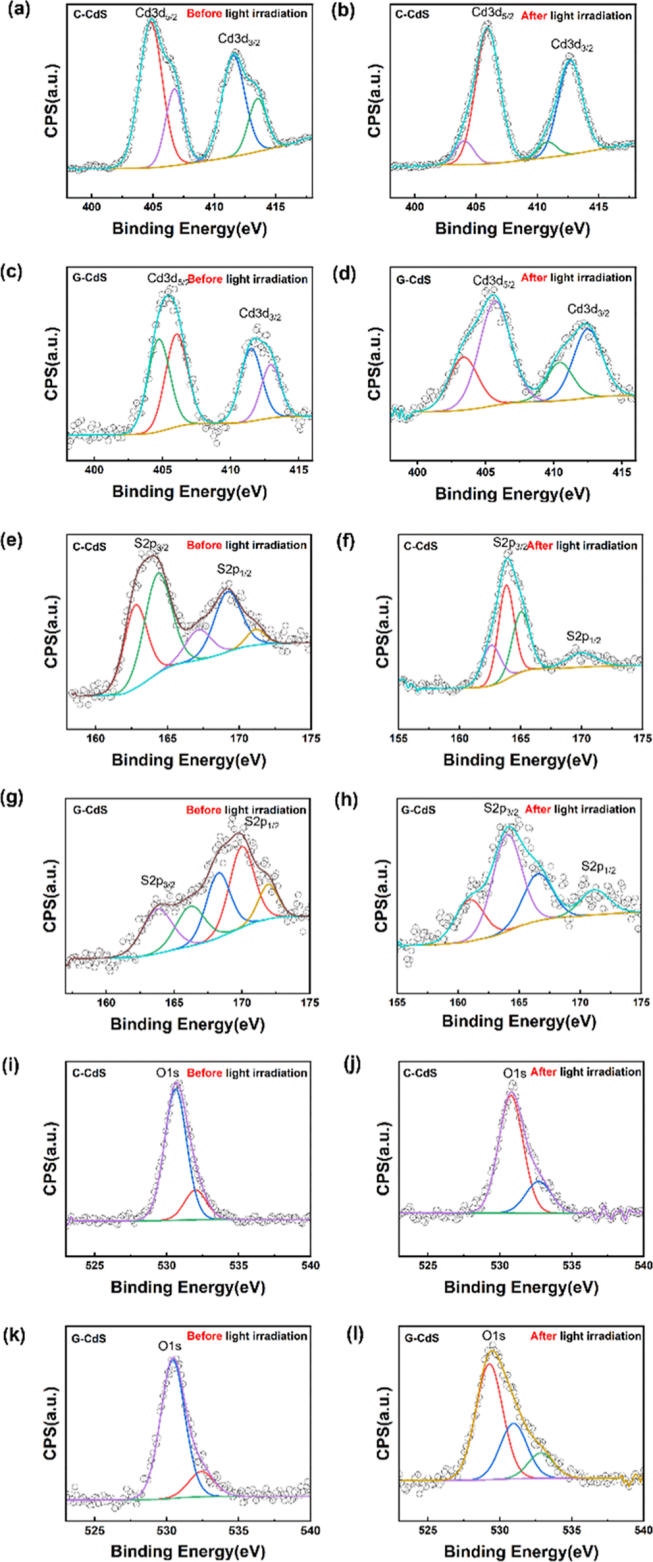
XPS results of C-CdS and G-CdS before and after PC reactions. Core spectra of C-CdS: (a,b) Cd 3d, (e,f) S 2p, and (i,j) O 1s before and after the PC reaction. Core spectra of G-CdS: (c,d) Cd 3d, (g,h) S 2p, and (k,l) O 1s before and after the PC reaction.

X-ray photoelectron spectra of C-CdS and G-CdS QDs before and after the PC reactions were recorded to understand the influence of polyphenol/chlorophyll moieties that are found to stabilize and protect the G-CdS QDs from photocorrosion and thereby enable enhanced photocatalytic efficiency. As observed from Figure 10a, the Cd (3d_5/2_) and Cd (3d_3/2_) peaks centered at ∼405 and ∼412 eV, respectively, for C-CdS QDs are in line with those reported in the literature.^68,69^ However, a positive shift in the binding energy is observed in the case of G-CdS QDs, which could be attributed to the presence of polyphenol/chlorophyll moieties. Such positive shifts in the peaks have been previously reported for CdS supported on graphene oxide.^70^ Further analyzing these peaks with Gaussian functions facilitates a detailed understanding of their chemical environment. The predominant unusual Cd 3d_5/2_ peaks of C-CdS observed at 404.6 eV (Figure 10a) could be attributed to either Cd(OH)_2_ or CdO_*x*_, wherein a thin hydroxide or oxide layer is typically formed on chemically synthesized CdS nanoparticles as reported by Wakerley et al.,^71^ who understood that the thin CdO_*x*_ oxide layer on the CdS surface protected it from photocorrosion. However, the Cd 3d_5/2_ peaks (Figure 10b) disappeared after the PC reaction, implying that the surface oxide layer is not sustainable under light irradiation. The Cd 3d_3/2_ peaks (Figure 10b) showed a broadening feature at 411.3 and 413.6 eV. However, only one peak appeared at 412.6 eV after the PC reaction, which indicates that Cd^2+^ ions could leach from the C-CdS catalyst to the electrolyte, and the same was observed through the PC cycle test (Figure 6c) and zebrafish embryos as the edema effect (Figure 9b).

Interestingly, the Cd 3d_5/2_ of G-CdS (Figure 10c) exhibits a dual peak (404.6 and 406.7 eV) with a center at ∼405.3 eV, which retains a similar position of 405.5 eV after the PC reaction. However, the Cd 3d_3/2_ peaks of G-CdS (Figure 10d) are observed to be slightly shifted from 411.2 eV to a higher binding energy of 412.3 eV after the PC reaction. Unlike C-CdS, the Cd^2+^ atoms in G-CdS are observed to be relatively robust, which could be attributed to the chlorophyll/polyphenol moieties coated onto G-CdS that enhance the stability.

The sulfur peaks of C-CdS observed at 163.9 eV (S 2p_3/2_) and 169.3 eV (S 2p_1/2_) (Figure 10e,f) were observed to get weaker after the PC reaction which is suggestive of photocorrosion owing to which the S^–^ species (and in turn the Cd^2+^) could leach into the electrolyte. On the other hand, the sulfur peaks in G-CdS exhibited a broadening nature (Figure 10g,h). Furthermore, the Gaussian fitting of the S 2p_3/2_ peak has multiple peaks at 163.7, 165.9, 168.3, 169.8, and 171.8 eV, which may correspond to unbound sulfur with thiol, thiophene, sulfonyl, sulfone, sulfonate, and so forth, resulting from a strong interaction between sulfur molecules of the CdS and organic species of tea leaf extract (polyphenol/chlorophyll).^72−74^ For instance, the presence of chlorophyll/polyphenol moieties could be attributed to the positive shift in the binding energy peaks in the core energy spectrum of S 2p, which has been observed in the literature when CdS nanoparticles were integrated with *N*-polyaniline and graphene oxide.^75^ However, the limited results make it difficult to understand the underlying mechanism of sulfur species interacting with tea leaf moieties. After the PC reaction, these peaks are observed to be shifted from their original position, indicating that sulfur species interacting with tea leaf moieties may be slightly modified.

Further analyzing the oxygen environment (O 1s) sheds light on the surface oxidation at C-CdS and G-CdS. The O 1s spectra observed in Figure 10i,k, for both G-CdS QDs and C-CdS QDs at ∼533 eV indicate the oxygen-containing groups in CdS, consistent with those reported in the literature.^76^ The predominant peaks observed from C-CdS (Figure 10i), around 530.6 and 532.0 eV, are assigned to oxygen atoms bonding with the crystal lattice (Cd–O) and a hydroxyl group (OH) adsorbed on the surface, respectively. As discussed earlier, it could be attributed to the possible oxidation of the CdS surface. Similar behavior is observed in G-CdS QDs (Figure 10k). Figure 10j,l reveals the effect of surface oxidation in C-CdS and G-CdS QDs after the PC reaction. Despite surface oxidation, C-CdS is observed to suffer from photocorrosion issues. However, the organic moieties coated onto G-CdS QDs being relatively robust is observed to protect the core CdS QDs from photocorrosion and these results are in line with the zebrafish embryo test discussed in Figures 7–9.

## 4 Conclusions

In conclusion, a cost-effective strategy for synthesizing G-CdS QDs with a biofunctional shell layer with chlorophyll/polyphenol moieties employing a cheap and abundantly available tea leaf extract is reported. Interestingly, the as-synthesized G-CdS QDs not only exhibited excellent photocatalytic activity in the degradation of MB dye molecules but also exhibited enhanced photocorrosion stability in comparison to chemically synthesized C-CdS QDs. Enhanced visible-light absorption, emission intensity, and photocatalytic activity of G-CdS QDs were attributed to the heterojunction formed between CdS QDs and polyphenol/chlorophyll molecules, which effectively inhibited the recombination of the photogenerated charge carriers and prevented photocorrosion. Surprisingly, the zebrafish embryos treated with G-CdS QDs, even at a moderately higher concentration, did not kill the organisms by virtue of the biocompatible polyphenol/chlorophyll shell layer. The enhanced survival rate of zebrafish embryos with minimal malformations treated with G-CdS QDs indicates that the chlorophyll/polyphenol moieties protected the CdS QDs from photocorrosion and prevented the leaching-out of the Cd^2+^ ions. This argument was verified by XPS measurements. It reveals that the chlorophyll/polyphenol moieties have strong interaction with sulfur atoms of CdS which protect from photocorrosion. Therefore, the green synthesis of nanostructured materials can surprisingly produce multifunctional effects that could enhance the overall physicochemical properties and enable biocompatibility that is favorable for various applications. Furthermore, the detailed nanotoxicity impact analysis using the zebrafish embryo model reported in this study can help researchers understand the toxic nature of small-sized nanoparticle photocatalysts and enable them to plan such studies to protect the environment from secondary pollution caused by the toxic ions that may leach out of photocatalysts.

## Supporting Information Available

Microscopic images of embryos treated with C-CdS QDs at 10 and 30 μg/mL and embryos treated with G-CdS QDs at a concentration of 10 and 30 μg/mL, observed at 24, 48, and 72 hpf, respectively (PDF)
