# Supplementary material for: Biofunctionalized CdS Quantum Dots: A Case Study on Nanomaterial Toxicity in the Photocatalytic Wastewater Treatment Process
Source: ACS Omega. 2023 May 22;8(22):19413–24. doi: 10.1021/acsomega.3c00496 (PMC10249079; doi:10.1021/acsomega.3c00496)
Supplement: Supplementary file 1 — ao3c00496_si_001.pdf [file ao3c00496_si_001.pdf]

Supporting Information

Bio-Functionalized CdS Quantum Dots: a case study on nanomaterials toxicity in photocatalytic wastewater treatment process

Kavitha Shivaji [1], Kishore Sridharan [2], D. David Kirubakaran [3], Jayaramakrishnan Velusamy [4], Seyedeh Sadrieh Emadian[5], Satheesh Krishnamurthy[5], Anitha Devadoss [6], Sanjay Nagarajan [7], Santanu Das [8] and Sudhagar Pitchaimuthu [9,*]

[1] Department of Biotechnology, K. S. Rangasamy College of Technology, Tiruchengode 637215, India.

[2] Department of Nanoscience and Technology, School of Physical Sciences, University of Calicut, Thenhipalam 673635, India.

[3] Department of Physics , K. S. R College of Arts and Science for Women, Tiruchengode-637215, India

[4] Department of Chemical Engineering and Biotechnology, University of Cambridge, Philippa Fawcett Drive, Cambridge CB3 0AS, UK.

[5] School of Engineering and Innovation, The Open University, UK.

[6]Institute of Biological Chemistry, Biophysics and Bioengineering (IB3), School of Engineering and Physical Sciences, Heriot-Watt University, Edinburgh EH14 4AS, UK.

[7]Department of Chemical Engineering, University of Bath, Bath BA2 7AY, UK.

[8] Department of Ceramic Engineering, Indian Institute of Technology (BHU), Varanasi 221005, India.

[9] Research Centre for Carbon Solutions, Institute of Mechanical, Processing and Energy Engineering, School of Engineering and Physical Sciences, Heriot-Watt University, Edinburgh EH14 4AS, UK.

[*] Correspondence: S.Pitchaimuthu@hw.ac.uk

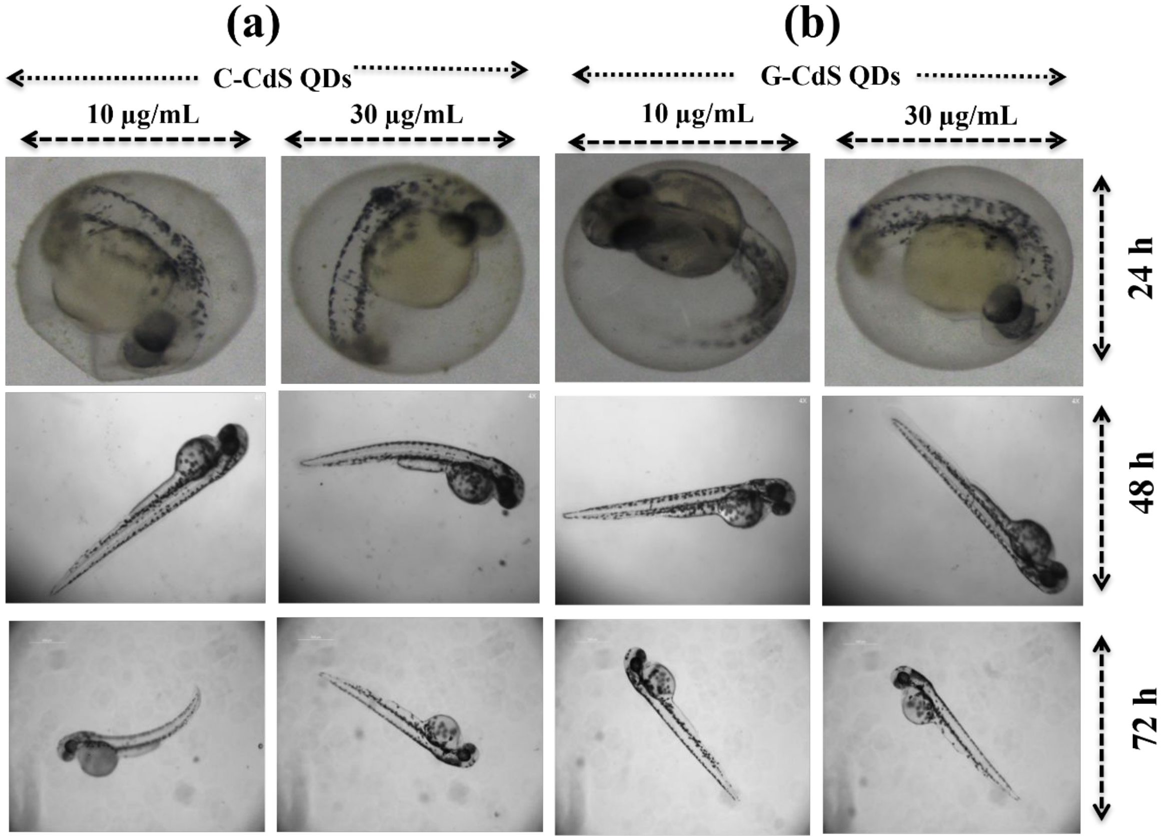

Figure S1. Microscopic images of (a) Embryos treated with C-CdS QDs at 10 and 30 µg/mL and (b) Embryos treated with G-CdS QDs at a concentration of 10 and 30 µg/mL, observed at 24, 48, 72 hpf, respectively.
